# The Moral Influence of the Study of Practical Anatomy

**Published:** 1850-09

**Authors:** Geo. Putnam

**Affiliations:** Sterling


### EDITORIAL DEPARTMENT.

---

*The Moral Influence of the Study of Practical Anatomy. Rev. Dr. Putnam and the Medical Profession.*

In the late effort to obtain a commutation of sentence in the case of Dr. Webster, the Rev. Dr. Putnam, the spiritual adviser of the prisoner, and his advocate before the Governor and Council, speaking in extenuation of the mutilation of the remains of the murdered man, used the following language:—

"That he could devise such a plan of disposing of the body, and then go through it with all the horrid and disgusting operations incident to it, has produced in all minds an impression unfavorable to his character. He cannot complain that it should. Nothing can ever remove that impression."

"There is but one word of abatement; and because there is no other, it ought to be remembered, viz.: that, to a man of medical education, a lifeless body has not the sacredness which it has to others: to him it is a mere subject to be handled and cut with no more awe or sensibility than the butcher has in dealing with the meat of the shambles. To the habitué of a medical college, the process of dismemberment is associated with the jokes and levities of student life and conversation; and can never be to him the same thing that it would be to another person of no greater moral feeling or principle."

Error and truth, in almost every human production, are more or less blended. Seldom is a truth uttered without some error, or an error without some truth. In the above quotation there is both truth and error; but the general impression left on the mind of the reader is, without a doubt, both unjust and ungenerous toward the medical profession. So it was considered by medical gentlemen in the city where the remarks were made; and in the Boston Daily Advertiser we find an article, written with much ability and fervor, vindicating the medical profession from the imputations which the quotation appeared to convey, and claiming for the study of anatomy as much exemption from *brutalizing* influences, as pertains to theological pursuits. We would copy the article were it not for its length, and we should do injustice to the writer by the publication of a fragment only of it.

In the same paper, of a subsequent date, the Rev. Dr. Putnam communicates a reply to the article just referred to; and we have seldom read a production which evinced a more candid and manly spirit. As it is short, we shall copy it entire, with the conviction that its perusal will afford our readers the same pleasure which we have derived from it. Under excited feelings, and with the mind bent on a particular object, language may inadvertently be used expressing far more, and perhaps admitting of a widely different construction from that which the author intended to convey. But, unfortunately, persons are not always ready to see the error into which they have been betrayed, and make amends for even an involuntary injustice. To such persons the careful consideration of the letter by Dr. P. may be properly commended.

**For the Boston Daily Advertiser.**

Mr. Editor,—In the Daily Advertiser of the 11th (Aug.) inst., a correspondent has offered some strictures on a brief passage taken from the remarks lately made by me to the Committee of the Executive Council in support of Dr. Webster's petition for a commutation of his punishment. He thinks the passage injurious and ungenerous towards the medical profession. Other physicians—friends, whose candor and kindness I cannot doubt—have intimated the same thing to me, though without the bitterness and severity of your correspondent. I am, therefore, constrained to believe, that I have unwittingly done injustice to others and to myself, in what I said in that connection. I perceive the infelicity of my language and its liableness to be misunderstood. Instead, therefore, of defending it word for word, I will endeavor to state now what I really did mean to say, and what I did not.

I was suggesting a slight extenuation—all that it admitted of—of Dr. Webster's conduct in disposing of the dead body of Dr. Parkman. His familiarity with the dissecting-room, I thought, must have prepared him to dismember the body, without the nervous horror and physical faintness that would be felt by another man of the same moral grade, but a stranger to medical dissection. I did not mean that the study of anatomy would make a man any more likely to commit a homicide; but I suppose I may say that *if* a man practised in anatomy *should* commit a homicide, with or without malice, and *if* he should decide upon the concealment of the body as necessary or expedient, the bare process of dismemberment would not be so revolting, so impossible a thing to him, as to another of the same degree of moral sensibility and principle. The surgeon or anatomist, of the tenderest feelings, can operate upon a human body, living or dead, with a steadiness of nerve and a cool self-possession of mind, which is only to be had by practice. What would become of the beneficent art of surgery, and the noble science of anatomy, if he could not? I could not make an incision into a human body, living or dead, without fainting away; that fact does not prove me to be humane or moral. The beloved and trusted physician of my family could do it without a qualm. Can any body suppose that I consider that power, which he has acquired by practice, as derogatory to his humanity or his virtue? the blessed power by which he relieves the suffering and prolongs the life of me and mine!

That the study of medicine, with the anatomical investigations so essential to it, is "demoralizing" in its influence on character, is no sentiment of mine. I never said it nor thought it; and I should not suppose it would be inferred from what I did say, on the occasion referred to. On the contrary, unless I have been singularly fortunate in my medical associations, physicians, as a class, are rather remarkable for humane feelings and tender sympathies. There are among them, certainly, as many men whom I love and revere for all that is most exalted and beautiful in human character, as in my own profession, or any other profession or calling whatever. And it has never yet occurred to me to imagine, that their ability to operate on a living body, or to dissect a lifeless one, with a courage, a steadiness of hand and composure of feeling, which I could not command, is any disparagement of their religious character, or their humane and social virtues.

I fully concur with your correspondent in all he has said, so well, of the nobleness and dignity of anatomical science, and the importance of the medical profession. I only regret, that, either through my fault or his own, he should see fit to place his sound and eloquent remarks in sharp antagonism with me and my words—words spoken in another connection, for another purpose, and with no idea of such a bearing as he finds for them. I hold the medical profession in as high honor as he does—in as high honor as I hold my own, or any other. I have no prejudices, *narrow* or broad, against it, or the admirable sciences which underlie it. I hold Physiology to be as legitimate a science as Theology,—indeed, a splendid branch of it. I assume for clergymen no superiority in virtue, or the sentiments and sympathies of humanity, over physicians. I agree with your correspondent, and am happy to confirm his remark from my own observation, that the physician enters the chamber of sickness, and the scenes of pain, death, and sorrow, with as soft a tread, as gentle a voice, and with as warm a current of affection in his heart, as the clergyman.

The physician and the clergyman, in many of their duties, find their paths run side by side, through scenes of anguish and woe. Each has his part to do. There is no rivalry, and no occasion for mutual disparagement or distrust. They should honor and help one another, as brethren and fellow-laborers in the noble and blessed service of humanity. If the tie that should unite them has been in any degree weakened by any word of mine, honestly misunderstood, or carelessly perverted, I wish that word recalled, as false to my thought, and justified by no feeling that I ever for a single moment entertained.

GEO. PUTNAM.

STERLING, July 12, 1850.

